# Compensation

**Published:** 1866-12

**Authors:** 


### COMPENSATION.

All the forces and events of the universe are unmistakably regulated by the law of distributive justice. Since the Morphological conception of the universe, the doctrine of evolution has taken the place of the notion of a mechanical creation, and the late discoveries in the world of science, have shown that *forces* are correlative and equivalent—e. g. given —so much heat, an equivalent amount of motion, light, electricity or magnetism may be evolved from it.

The idea of the correlation and equivalence of forces, is the inductive manner of stating that an eternal law of justice rules in the physical, mental and moral worlds. The late experiments in science establish a correlation and equivalence between forces. Thus, the fall of 772 lbs. through one foot raises one pound of water one degree Fahrenheit. The proved relation in amount between the affinities of combining bodies, and the heat evolved during their combination, the quantitative connection between chemical action and voltaic electricity, already established—the experiments of Faraday implying that a specific measure of electricity is disengaged by a given measure of chemical action—the demonstrated equivalence between the amount of heat generated and water converted into steam—the known expansion of steam under the influence of each additional degree of heat, all render it certain that among the various forms of force the quantitative relations are fixed.

And hence the fall of the sunbeams on the earth finds its correlative and equivalent in the rise of vapor and the consequent fall of the snows, rains and rivers to the earth and into the sea.

The silent rush of the planets through the heavens, is but the transformed molecular motion of the original photosphere of the sun. The world of forces obeys the laws of addition, subtraction, multiplication and division; and mind itself is a force, or rather the aboriginal source of all forces. Grant a

God—an eternal, infinite mind—and this correlation and equivalence of forces is but the outer physical *ensemble* of the inherent laws of Divine reason—of eternal retributive justice. This law holds between physical and vital, and vital and mental forces. The more mind a man exhibits, the more blood he burns up or exhausts; and the more blood he exhausts, the more food, heat and air he requires. And it holds also between the individual and society—between man and men. This law secures that we get from our fellows what we pay for, no more, no less. It secures that the quack shall be quacked at last, and the longer the credit given, the heavier the interest which will be ultimately required. If he seems to triumph for a time, it is only that his falsehood may lift him to the verge of his ambition in order to secure his more complete ruin when he falls. And, then, his social ruin reduces him to his true level from which he can, if he will, rise to a more elevated station among his fellows. To illustrate: The dentist if he be merely an artist—merely a worker in *contraband* rubber and mineral teeth—a modern Tubal Cain, and not also a teacher of his patient—instructing how to save teeth from decay, may get his full compensation in dollars for his artifice, but he will not command that utter trust, that moral confidence, that sublime fraternity which is the true compensation for the work of the soul. There is a higher form of this law of compensation than that which for a certain expenditure of professional skill, shall fill one's tills with money.

All exist for moral, as well as for pecuniary benefits, else they are not men.

The professions become grand, sacred, blessed, in just the ratio of their actual power to benefit the world. There is something of a practical immorality in the present relations of the professions to the people. The physician thrives on sickness, the lawyer on quarrels, and the dentist on decayed teeth. "Let these professions teach the people how to *preserve* health, to *avoid* difficulty, and to *prevent* the decay of the teeth, perfectly, and our business would be destroyed, I hear you say. Very well, which is first and paramount, man, or his diseases and the consequent wealth of the professions? Does man exist merely that doctors, lawyers and dentists may thrive? Or do the professions arise to save, to preserve and elevate man? If one affirm the first, all moral dignity and worth depart the professions. But if the second be affirmed, all the professions become the physical and moral regenerators of society—the grandest moral element enters into their career and function, and the compensation for such professional service, is a gradually regenerated, beautified, lofty-minded society. Let the people pay us to teach them how to save themselves from the ills incident to a perverted civilization, and we are at once in harmony with all the great interests of mankind, and the moral laws of God. The compensation for such relationship is measureless happiness and blessedness.

Now it is the financial interest of the physician to have everybody sick, of the lawyer to have everybody in a quarrel, and of the dentist that the peoples' teeth may rapidly decay. Add the moral law of compensation and nobody likes the members of either profession named. The people look upon us as "necessary evils."

At once, let us shift our relations, so that we become principally teachers, and incidentally only manipulators, &c., and while the long continued sins of our ancestors will secure us plenty of patients for some generations to come, we shall yet lay the foundation for a broader and higher professional character, at once consistent with all the laws and interests of man and of society.

Nor is this all since the moral and spiritual laws are primordial, and obedience to them secures the highest possible inspiration to genius, talent and intellect. "Talent invariably sinks with character," it must therefore rise with character. Elevate the professions into harmony with the primordial code of nature, which recognizes exact compensation for every act or failure to act—for every mental effort and aspiration for the highest good, and our professional ranks will be ablaze with genius, with talent and new discoveries. New ideas, a new society will arise upon the world. Great aims alone can quicken genius, and attract it into the centre of social power.

"Moreover, a moral compensation reacheth to the secrecy of thought;
For if thou wilt think evil of thy neighbor, soon shalt thou have him for thy foe:
And yet he may know nothing of the cause that maketh thee distasteful to his soul—
The cause of unkind suspicion, for which thou hast thy punishment:
And if thou think of him in charity, wishing or praying for his weal,
He shall not guess the secret charm that lured his soul to love thee."

